# Oxidative Stress and its Implications for Future Treatments and Management of Alzheimer Disease

**Published:** 2010-09

**Authors:** Timothy A. Clark, Hyun Pil Lee, Raj K. Rolston, Xiongwei Zhu, Michael W. Marlatt, Rudy J. Castellani, Akihiko Nunomura, Gemma Casadesus, Mark A. Smith, Hyoung-gon Lee, George Perry

**Affiliations:** 1 *Department of Pathology, Case Western Reserve University, Cleveland, Ohio, USA;*; 2 *Geisinger Medical Center, Danville, Pennsylvania, USA;*; 3 *Swammerdam Institute for Life Sciences – Center for Neuroscience, University of Amsterdam, Amsterdam, The Netherlands and Marie Curie Early Stage Training Program – NEURAD Graduate School, Göttingen, Germany;*; 4 *Department of Pathology, University of Maryland, Baltimore, Maryland, USA;*; 5 *Department of Neuropsychiatry, Interdisciplinary Graduate School of Medicine and Engineering, University of Yamanashi, Chuo, Yamanashi, Japan;*; 6 *Department of Neurosciences, Case Western Reserve University, Cleveland, Ohio, USA;*; 7 *UTSA Neurosciences Institute and Department of Biology, College of Sciences, University of Texas at San Antonio, San Antonio, Texas, USA*

**Keywords:** apolipoprotein E, copper, free radical, iron, lipid peroxidation, oxygen species, reactive, vitamin E

## Abstract

Oxidative imbalance is one of the earliest manifestations of Alzheimer disease (AD) actually preceding the classic pathology of amyloid β deposits and neurofibrillary tangles. Clinical trials examining antioxidant modulation by a number of global interventions show efficacy, while simple supplementation has limited benefit suggesting complexity of multiple contributing factors. In this review, we highlight new insights regarding novel approaches to understanding and treating AD based on holistic views of oxidative balance including diet.

## INTRODUCTION

Alzheimer disease (AD) is the most common neurodegenerative disease among the elderly. Common pathological hallmarks which manifest in AD include senile plaques of amyloid-β (Aβ) aggregates and neurofibrillary tangles (NFTs) comprising of paired helical filaments (PHFs) of tau protein (1). Other irregularities include neuronal and dendritic loss, accumulation of neuropil threads and dystrophic neurites, and atrophy of the brain (2). While they are useful in diagnosing AD, pathological hallmarks do not provide insight towards understanding the pathogenesis of the disease (3).

In this review, we emphasize studies on the connection between oxidative stress and AD pathology, recent approaches to the prevention and treatment of AD.

The free radical theory of aging suggests that oxidative imbalance is a major player in the degeneration of cells (4, 5). With age as the primary risk factor for AD, free radicals have been implicated as a possible origin for AD. Oxidative stress has been defined as a breaching of the intracellular capacity for removing free radicals, leading to modification of DNA, lipids, polysaccharides, and proteins (6-8) as well as the altered homeostatic balance resulting from increased antioxidant defenses. Markers for oxidative damage, including carbonyls, hydroxynonenal, and malonaldehyde are increased in AD and essentially every form of antioxidant stress response has been reported increased in AD.

Free radicals are molecules which carry an unpaired electron, making them highly reactive and ready to gain an electron in any way possible. Superoxide, hydroxyl, nitric oxide, alkoxyl, and peroxyl radicals are the most common free radicals within cells. Other molecules are not free radicals but can lead to the production of free radicals through various chemical reactions, such as hydrogen peroxide (H_2_O_2_), and peroxynitrite (ONO_2_). Free radicals and similar molecules are regularly classified collectively as reactive oxygen species (ROS) indicating their capacity to cause oxidative modifications within the cell. Because free radicals are unstable and highly reactive, they are kept at relatively low levels by detoxifying enzymes. ROS are a natural byproduct of the metabolic pathways of oxidative phosphorylation during cellular respiration. However, sometimes, the production of ROS can surpass the cell’s ability to remove them, resulting in the imbalance of oxidative homeostasis leading to oxidative stress (9).

Due to its elevated levels of peroxidizable fatty acids, high request for oxygen, and relative paucity of antioxidant systems, the brain is extremely sensitive to oxidative stress. Altered mitochondrial function, Aβ peptides, and the presence of trace metal ions such as iron and copper, have been identified as potential sources of oxidative stress (10-12). It is now understood that these three areas are not mutually exclusive. For example, Aβ may induce the production of ROS in the mitochondrial membrane causing subsequent oxidative damage in the early stages of disease progression. This has been shown in studies of AD patients as well as in transgenic mice overexpressing AβPP (11, 13-16). Suprisingly, redox-active transition metals collect in AD susceptible neurons (10) and, along with Aβ, can locally produce higher levels of ROS when around cytoplasmic H_2_O_2_ (17-19) leading to lipid and RNA oxidation (20). There are likely numerous mechanisms which cause oxidative stress to occur leading to dysfunctional neuronal responses in AD and the progression of the AD (21-23).

**Figure 1:**
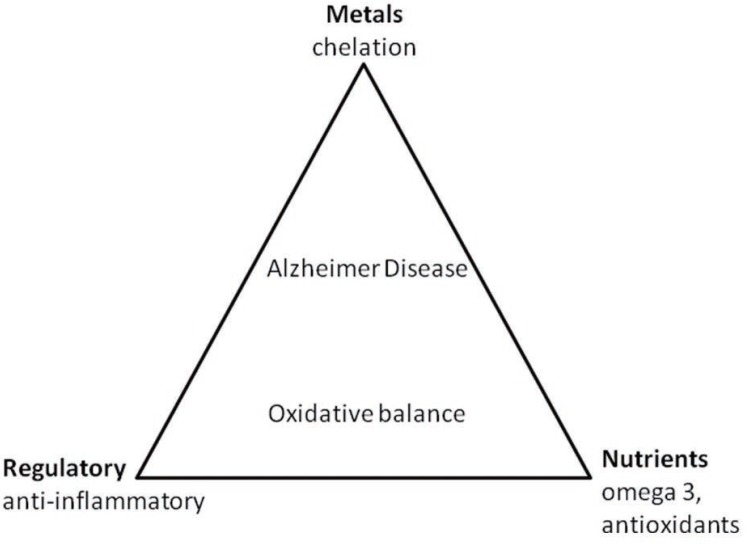
Therapeutic modulation of oxidative balance in Alzheimer disease can be accomplished by a triad of interventions.

Antioxidant trials include the examination of exogenous or endogenous compounds, which act to scavenge ROS, inhibit ROS formation, or bind metal ions needed for ROS generation. Natural antioxidants can be classified into two major groups: enzymatic antioxidants (e.g., superoxide dismutase, catalase) and nonenzymatic cellular molecules mimicking antioxidants (Figure 1). Some common nonenzymatic molecules include glutathione (GSH), ascorbate (vitamin C), α-tocopherol (vitamin E), β-carotene, uric acid, sodium selenite, melatonin, and plasma protein thiol.

Enzymatic antioxidants have cytoprotective effects in AD model systems yet have been shown to be reduced during the disease process (24). GSH reduces free radicals in vivo yet the level of GSH is decreased both in cortex and hippocampus of patients with AD (25-27). Vitamin E is the most effective nonenzymatic antioxidant within the cell membrane and is able to reduce lipid peroxidation (28).

While it has not yet been understood, having a healthy diet has been shown to decrease AD risk. An intake of dietary vitamin C and vitamin E have been associated with a decreased risk of the disease. This relationship, however does not exist for supplement only intake (29). Fish consumption and diets rich in Omega-3 fatty acids have also been correlated with a lower risk of AD development (30). High calorie and saturated fat diets show a relationship to increased AD risk (31). Further studies are needed to understand whole food intake and long term dietary pattern as they relate to cognitive decline.

Aβ is highly redox active and generates ROS in the presence of copper and iron (19, 32, 33). Because of the high levels of redox active metals in the brain during AD, chelation may be a possible form of therapy (10). Deferoxamine, a transition metal chelator, was shown to slow the rate of decline of daily living skills (34).

So far, no clinical study has succeeded in fully stopping the progression of AD. With increasing understanding of AD pathogenesis, it is clear that the focus of AD therapy will move to targeting oxidative stress. Ongoing studies are being done in the United States and Europe to better characterize between mild cognitive impairment and AD patients with the hope of improving clinical trials. The Alzheimer Disease Neuroimaging Initiative is currently underway between the NIH and private industry. This study will collect a wealth of data from MRI and PET scans, cognitive scores, and biomarkers, leading to more improved and more specific treatments. Understanding that the molecular mechanisms influential during disease progression should be the focus of treating AD, rather than the final pathological outcome (35), has put us another step closer to a world without AD.
